# Supplementary material for: Real-time observation of tetrapyrrole binding to an engineered bacterial phytochrome
Source: Commun Chem. Author manuscript; Available in PMC 2021 Nov 5. (PMC8570541; doi:10.1038/s42004-020-00437-3)
Supplement: Supplementary file 1 [file NIHMS1718943-supplement-Supplementary_Information.pdf]

Supplementary information for:

Real-time observation of tetrapyrrole binding to an engineered bacterial phytochrome

Yusaku Hontani[1,†], Mikhail Baloban[2], Francisco Velazquez Escobar[3], Swetta A. Jansen[1], Daria M. Shcherbakova[2], Joern Weißenborn[1], Miroslav Kloz[1,4], Maria Andrea Mroginski[3], Vladislav V. Verkhusha[2,5], and John T.M. Kennis[1,*]

[1]Department of Physics and Astronomy, Vrije Universiteit Amsterdam, Amsterdam 1081 HV, The Netherlands

[2]Departments of Anatomy and Structural Biology, Albert Einstein College of Medicine, Bronx, NY 10461, USA

[3]Institut für Chemie, Technische Universität Berlin, Sekr. PC 14, Straße des 17. Juni 135, Berlin D-10623, Germany

[4]ELI-Beamlines, Institute of Physics, Na Slovance 2, 182 21 Praha 8, Czech Republic

[5]Medicum, Faculty of Medicine, University of Helsinki, Helsinki 00290, Finland

[†]Current address: School of Applied and Engineering Physics, Cornell University, Ithaca, NY 14853, USA

*Correspondence and requests for the materials should be addressed to J.T.M.K. (email: j.t.m.kennis@vu.nl)

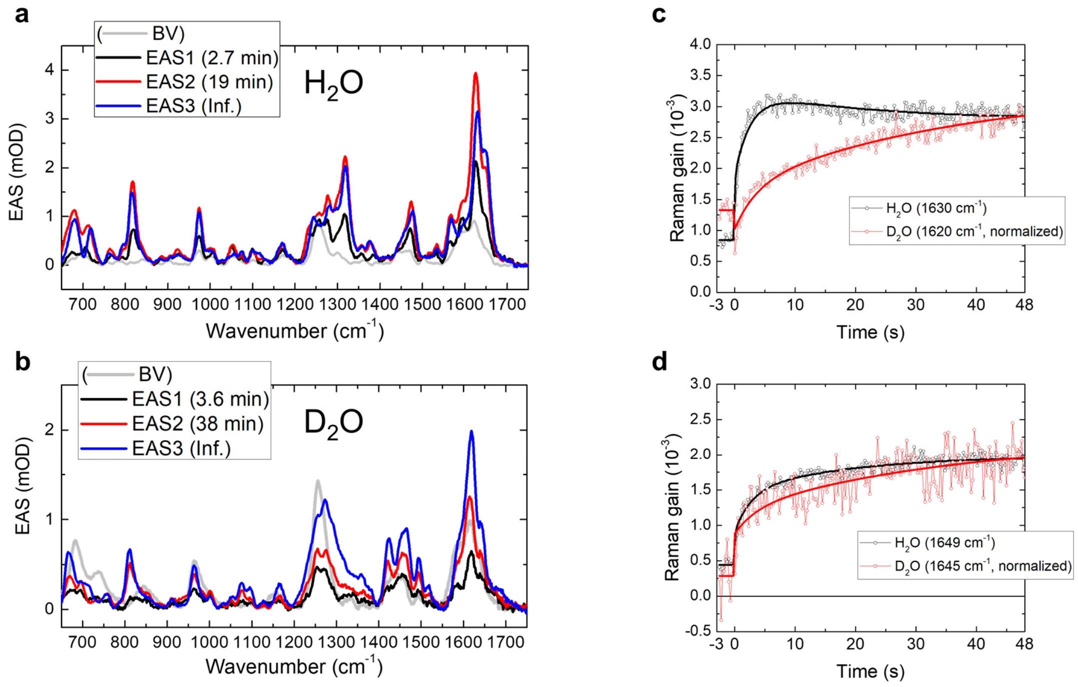

Supplementary Figure 1. Globally-fitted EAS and time traces of time-resolved Raman spectra of miRFP670/C20A. EAS obtained in (**a**) H_2O and (**b**) D_2O. Time traces at (**c**) 1630 cm^{-1} in H_2O (black) and 1620 cm^{-1} in D_2O (red), and (d) 1649 cm^{-1} in H_2O (black) and 1645 cm^{-1} in D_2O (red). The open dots show the raw data, and the solid lines show fitted time traces.

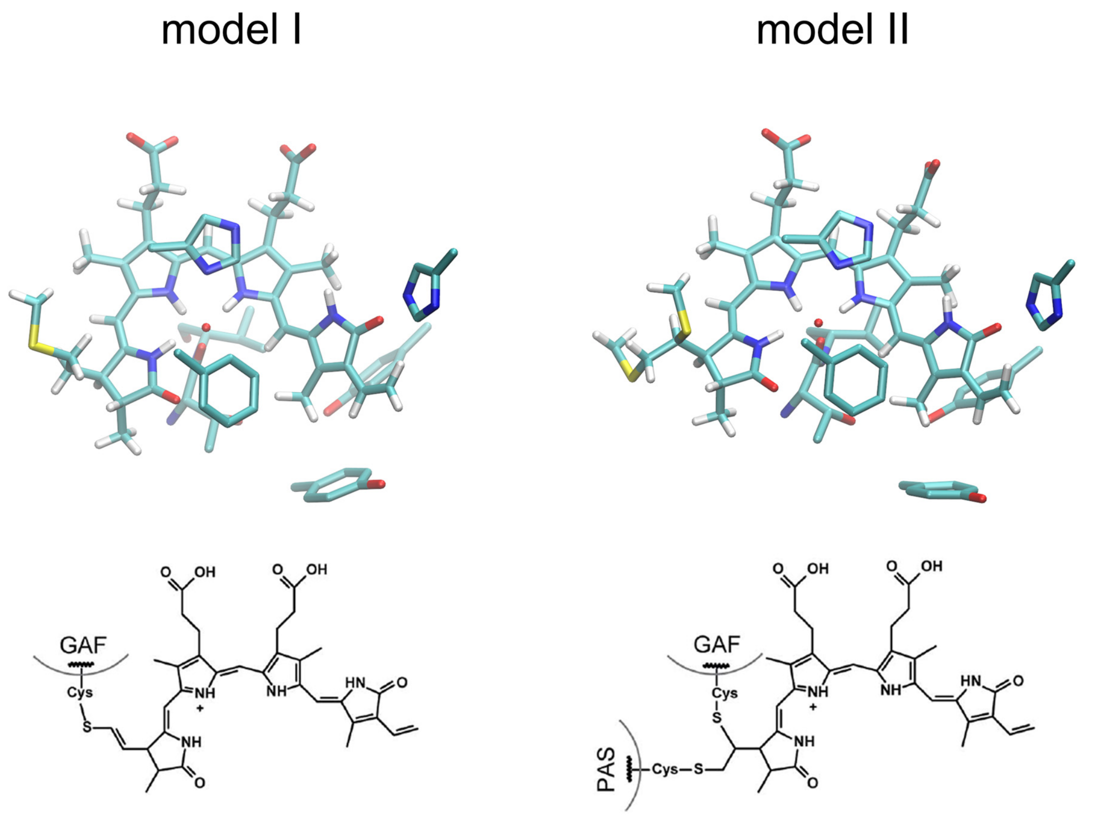

Supplementary Figure 2. Molecular models for QM/MM calculation. Based on the 3D structure (PDB entry: 5VIV[1]), two models where generated considering the two chromophore species detected in the electron density maps: model I, characterized by a single covalent bond between BV- $C3^2$ and the Cys253 of the GAF domain; model II, characterized by two covalent bonds between BV-$C3^1$ and Cys253 and between $C3^2$ and Cys20 from the PAS domain.

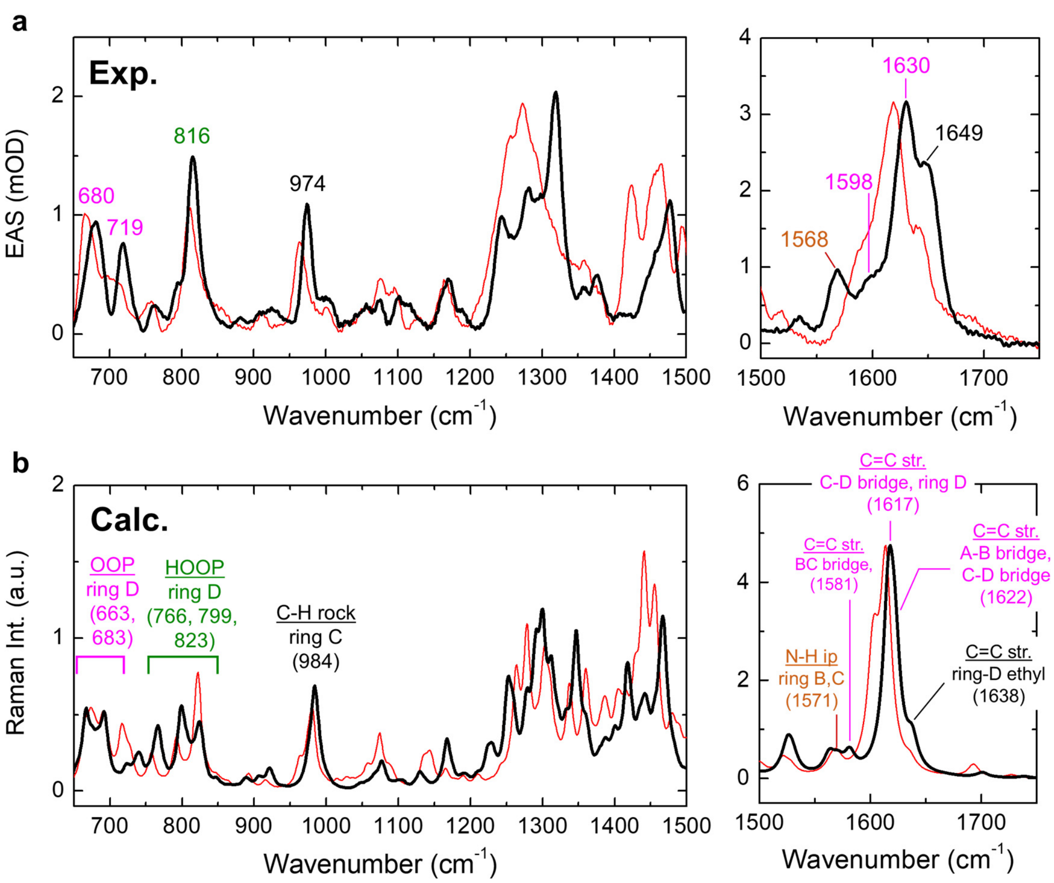

Supplementary Figure 3. Comparison of experimental and computational Raman spectra of miRFP670/C20A. (a) Third EAS of miRFP670/C20A from **Figures 2d–f and Supplementary Figure 2**. **(b)** QM/MM calculation of Raman spectrum of miRFP670/C20A. The black and red lines indicate data obtained in H_2O and D_2O, respectively.

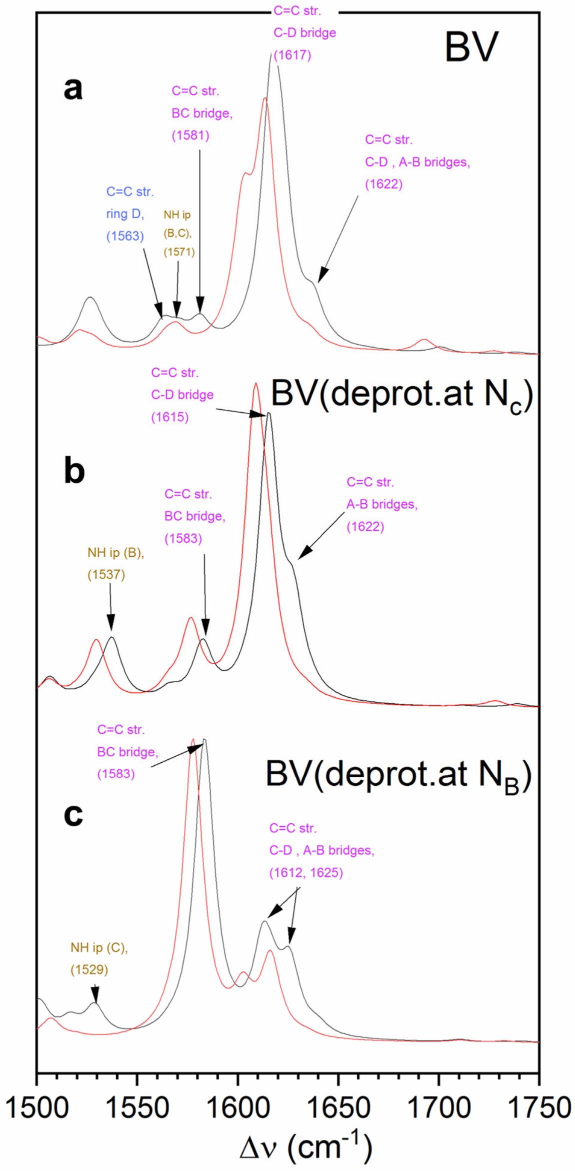

Supplementary Figure 4. Calculated Raman spectra of BV with protonated and deprotonated pyrrole rings. QM/MM calculation of Raman spectra of miRFP670/C20A (model I in Supplementary Figure 2) for **(a)** BV with four protonated pyrrole nitrogens, **(b)** BV with one deprotonated pyrrole nitrogen in ring C, **(c)** BV with one deprotonated pyrrole nitrogen in ring B. Black and red lines show spectra in H_2O and D_2O, respectively.

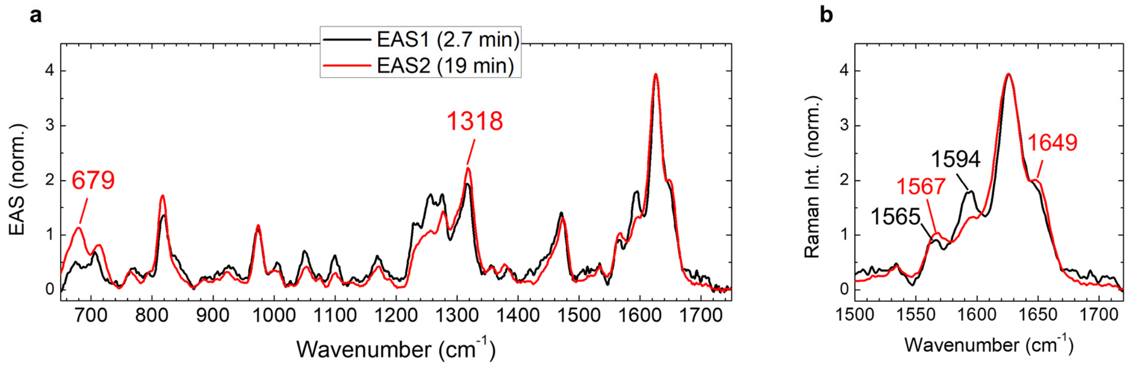

Supplementary Figure 5. Normalized EAS of time-resolved stimulated Raman spectra of miRFP670/C20A. (a) The Raman spectra at 650–1750 cm^{-1}. **(b)** Close-up Raman spectra at 1500–1720 cm^{-1}. The Raman spectra are normalized at 1626 cm^{-1}.

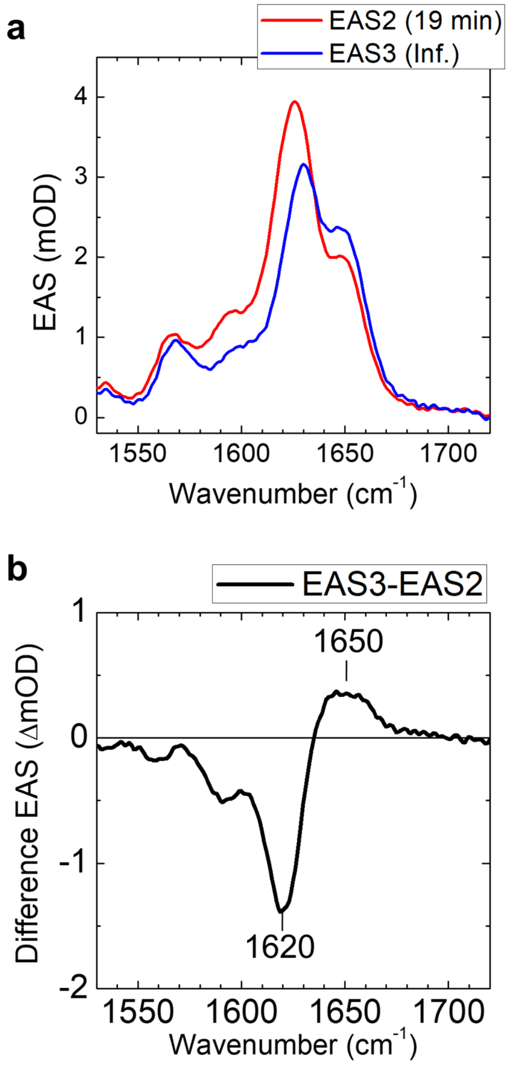

Supplementary Figure 6. Difference spectra of EAS of time-resolved stimulated Raman spectra in miRFP670/C20A at pH 8.0. (a) Evolution-associated spectra (EAS), reproduced from Figure 3f. The 2nd (red) and 3rd (blue) EAS are displayed. **(b)** A difference spectrum of EAS: the 3rd EAS minus the 2nd EAS.

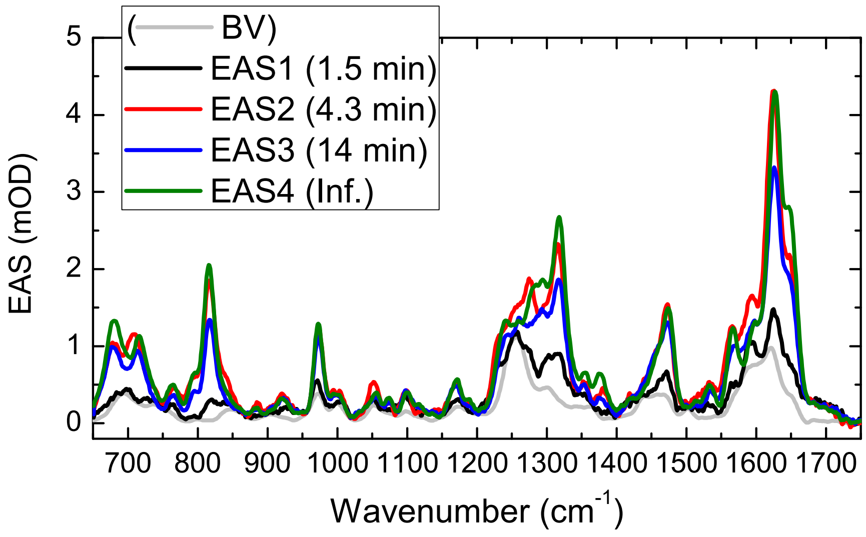

Supplementary Figure 7. Globally-fitted EAS of time-resolved Raman spectra of miRFP670 at pH 8.0.

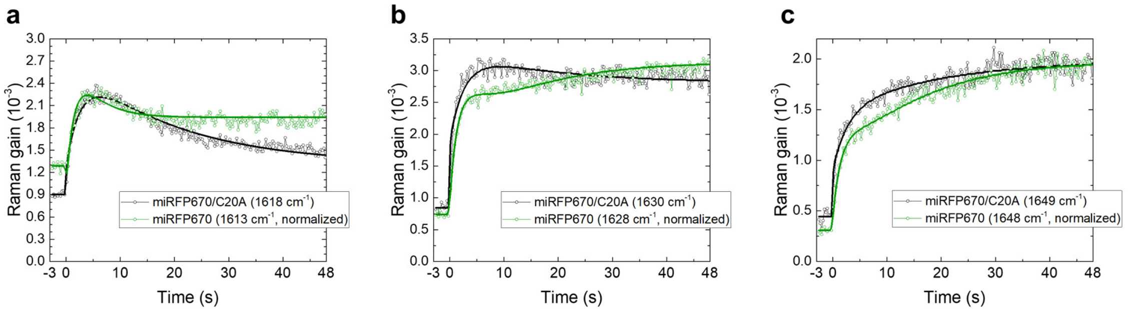

Supplementary Figure 8. Time traces of the Raman data in miRFP670 (green) and its C20A mutant (black) in H_2O. The open dots show the raw data, and the solid lines show the fitted time traces.

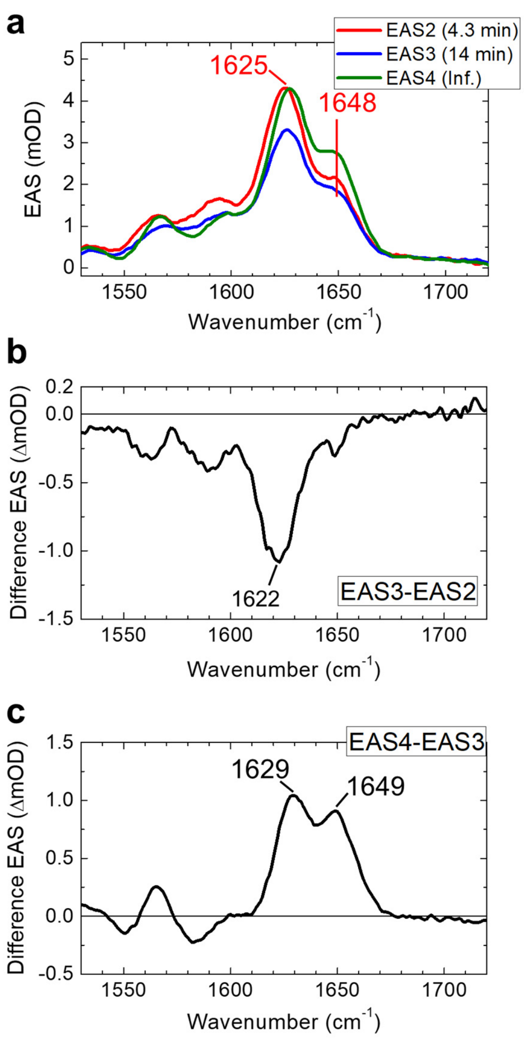

Supplementary Figure 9. Difference spectra of EAS of time-resolved stimulated Raman spectra in miRFP670 at pH 8.0. (a) Evolution-associated spectra (EAS), reproduced from Figure 4e. The 2nd (red), 3rd (blue), and 4th (green) EAS are displayed. **(b)** A difference spectrum of EAS: the 3rd EAS minus the 2nd EAS. **(c)** A difference spectrum of EAS: of the 4th EAS minus the 3rd EAS.

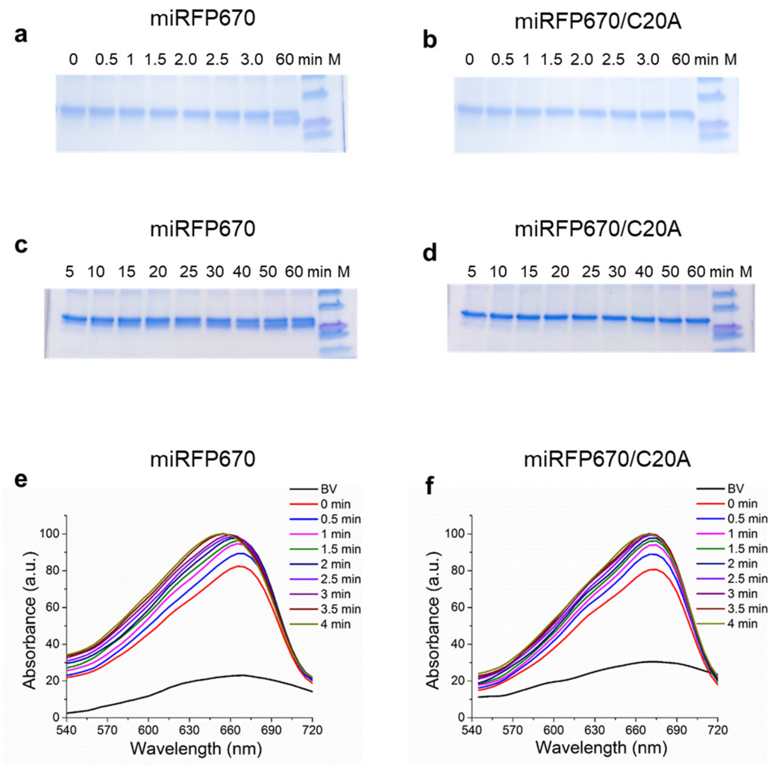

Supplementary Figure 10. Biliverdin assembly kinetics. SDS-PAGE protein gels of **(a,c)** miRFP670 and **(b,d)** miRFP670/C20A. Absorbance spectra at indicated time points of **(e)** miRFP670 and **(f)** miRFP670/C20A.

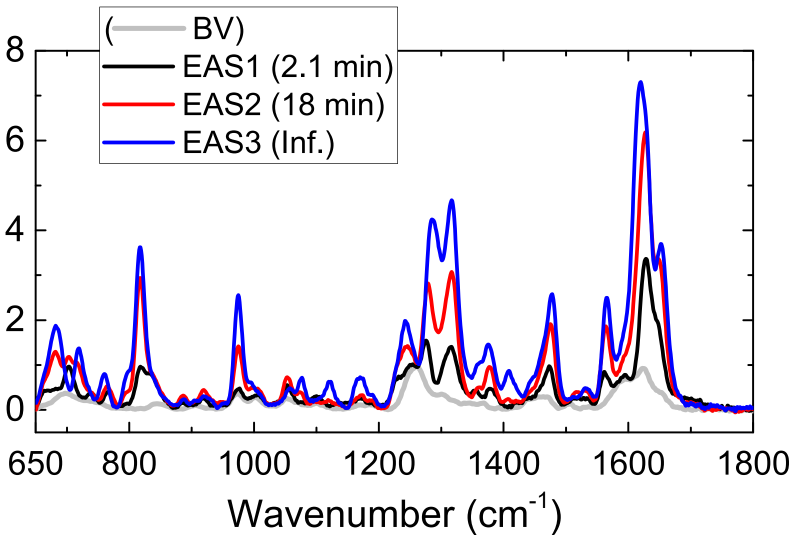

Supplementary Figure 11. Globally-fitted evolution-associated spectra of time-resolved Raman spectra of miRFP709 at pH 8.0. Evolution-associated spectra (EAS) with time constants of 2.1 min (black line), 18 min (red line) and nondecaying (blue line). The biliverdin (BV) stimulated Raman spectrum before addition of apoprotein is indicated with the grey line.

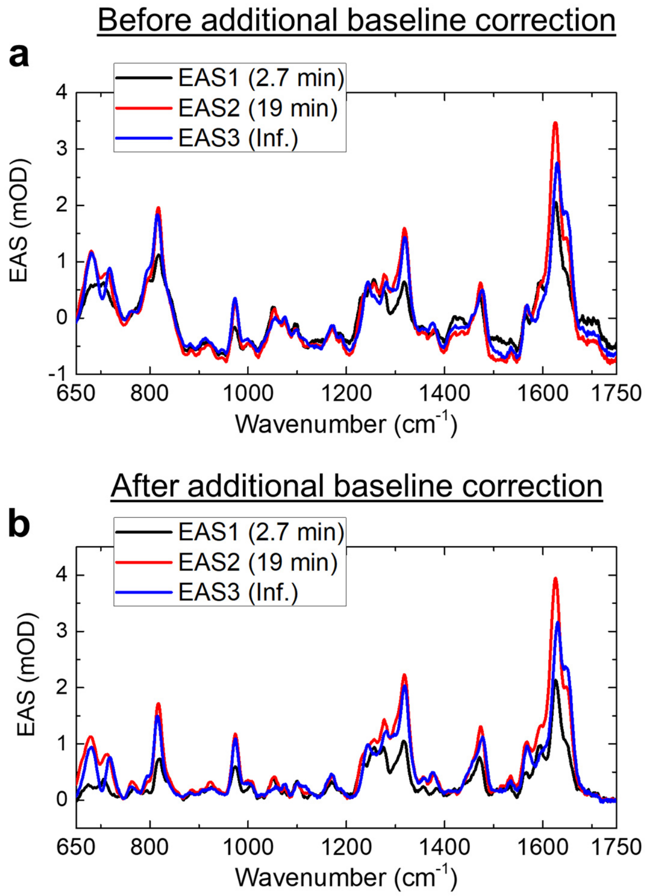

Supplementary Figure 12. Comparison of evolution-associated spectra of the stimulated Raman spectra of miRFP670/C20A in H_2O. Evolution-associated spectra (EAS) of **(a)** before and **(b)** after the additional manual baseline correction, as described in the methods.

Supplementary Table 1. Mode Assignment based on QM/MM calculation.

Model I (cm^{-1})	
1636.83	(20.26%) STRE C=C (Ring D Ethyl)
	(29.42%) STRE C=C (Methine Bridge C - D)
1622.26	(22.56%) STRE C=C (Ring D Ethyl)
	(8.40%) STRE C=C (Methine Bridge A - B)
	(16.28%) STRE C=C (Methine Bridge C - D)
1614.68	(20.94%) STRE C=C (Methine Bridge A - B)
	(7.01%) STRE C=C (Methine Bridge C - D)
	(9.01%) ROCK N-H (Ring A)
1583.82	(10.77%) STRE C=C (Methine Bridge B – C)
	(21.23%) STRE C-C (Methine Bridge B - C)
	(9.66%) ROCK N-H (Ring B)
	(11.08%) ROCK C-H (Methine Bridge B - C)
1575.95	(15.11%) ROCK N-H (Ring B)
	(41.47%) ROCK N-H (Ring C)
**Model II (cm^{-1})**	
1647.71	(22.03%) STRE C=C (Ring D Ethyl) -> 1
	(27.05%) STRE C=C (Methine Bridge Ring C - D)
1633.20	(6.21%) STRE C=C (Ring D Ethyl) -> 1
	(34.58%) STRE C=C (Methine Bridge A - B)
	(9.01%) ROCK N-H (Ring A)
1629.11	(18.19%) STRE C=C (Ring D Ethyl)
	(26.04%) STRE C=C (Methine Bridge C - D)
1585.49	(14.68%) STRE C=C (Methine Bridge B - C)
	(6.59%) STRE C-C (Methine Bridge B - C)
	(32.31%) ROCK N-H (Ring C)
1580.68	(15.54%) STRE C-C (Methine Bridge B - C)
	(20.93%) ROCK N-H (Ring B)
	(8.82%) ROCK N-H (Ring C)
1571.72	(9.19%) STRE C=C (Ring D Ethyl)
	(40.72%) STRE C-C (Ring D)
	(6.21%) ROCK N-H (Ring C)

References

1. Baloban, M. *et al.* Designing brighter near-infrared fluorescent proteins: Insights from structural and biochemical studies. *Chem. Sci.* **8**, 4546–4557 (2017).
